# Case Report: A playful digital-analogical rehabilitative intervention to enhance working memory capacity and executive functions in a pre-school child with autism

**DOI:** 10.3389/fpsyt.2023.1205340

**Published:** 2023-09-29

**Authors:** Sabrina Panesi, Marina Dotti, Lucia Ferlino

**Affiliations:** ^1^Institute for Educational Technology of the CNR, Genoa, Italy; ^2^David Chiossone Onlus, Genoa, Italy

**Keywords:** case report, working memory, executive functions, rehabilitative intervention, Autism Spectrum Disorder, preschool, app

## Abstract

**Background:**

Autism Spectrum Disorder (ASD) is often associated with deficits in Working Memory Capacity (WMC) and Executive Functions (EFs), as early as the first years of life. Research has shown that, even young children with ASD, WMC and EF deficits can be effectively addressed through interventions employing digital and/or analogical tools. Early intervention is important because executive dysfunction can negatively impact on the quality of life, both of children and their families. However, very few studies have been carried out involving intervention with pre-schoolers with ASD. To fill this gap, we developed an intervention that promotes pre-schoolers’ WMC and EFs by employing both digital apps and analogical playful activities. This study reports on the feasibility of this intervention, which was carried out in a rehabilitative context.

**Methods:**

A male pre-schooler diagnosed with ASD was engaged in a total of 17 intervention sessions, all held in a clinical context, over a nine-week period. Outcomes were measured using a battery of pre- and post-treatment tasks focusing on WMC, EFs and receptive language. The clinician who administered the intervention made written observations and noted any improvements in the child’s performance emerging from the digital and analogical activities.

**Results:**

The pre- and post-test scores for the cognitive tasks revealed qualitative improvements in the following cognitive domains: (a) WMC in the language receptive domain; (b) updating in WMC; (c) inhibition, specifically concerning control of motor response; (d) receptive vocabulary. Furthermore, when monitoring the child’s performance, the clinician noted improvement in almost all the playful activities. Particularly notable improvements were observed in interaction with the apps, which the child appeared to find very motivating.

**Conclusion:**

This study supports feasibility of a playful digital-analogical intervention conducted by a clinician in a rehabilitation context to promote cognitive abilities in pre-schoolers with ASD. Further studies are needed to establish whether the intervention’s effectiveness can be generalized to a broad sample of children with ASD.

## Introduction

Autism Spectrum Disorder (ASD) is a neurodevelopmental disorder characterized by impairments in social interaction, communication and sometimes stereotyped behavior [DSM-5; (1)]. Individuals with ASD constitute a heterogeneous group, with significant symptom variability, the presence/absence of comorbidities, including psychiatric comorbidities (2), impaired empathy in both cognitive and affective dimensions (3), and various types of cognitive difficulties (4).

In terms of cognitive abilities, ASD is often associated with deficit in working memory capacity (WMC) and Executive Functions (EFs). The term “Working Memory Capacity” (WMC) refers to a limited capacity system that allows information to be temporarily stored and manipulated (5). Miyake et al. (6) argue that WMC may rely on EFs, defined as a family of adaptive, goal-directed, top-down mental control processes (e.g., (7)). Further investigation (6, 8) has revealed that EFs comprise three main components: *inhibition,* the ability to suppress task-irrelevant cognitive processing and ignore salient yet irrelevant information; *shifting*, the ability to switch between different operations or levels of processing; and *updating,* the ability to encode, retain and monitor incoming information in working memory.

Recent literature reviews and meta-analyses reveal that, compared to individuals with typical development, those with ASD have impairments in WMC (9), inhibitory control (10), and cognitive flexibility (11). The construct of updating remains under-investigated, especially in the preschool period (12, 13). What’s more, the findings from the few studies that explore this EF component in autism have proven to be inconsistent (9).

Impaired WMC and EFs in individuals with ASD has a negative impact on self-regulation and daily functioning, especially concerning autonomy. Hence, it is important to enhance these components through cognitive interventions [e.g., (4)] administered as early as the first years of life.

Both digital and analogical-based interventions can be adopted to promote WMC and EFs in individuals with ASD. In a meta-analysis, Grynszpan et al. (14) highlighted the effectiveness of technology-based training for children with ASD. They establish that children find this type of training enjoyable and safe; it constitutes a secure environment in which errors have minimal consequences and therefore trigger less social anxiety and shame (15). Similarly, the meta-analysis performed by Pasqualotto et al. (4) analyses computerized and non-computerized training. They report growing evidence for the overall effectiveness of cognitive training as a tool to enhance WMC and EFs, particularly when activities are computer based.

It’s worth noting that most of the studies in these meta-analyses regard school-age children or older. Despite the importance of cognitive development at preschool age (16, 17), very few studies focus on rehabilitative intervention to enhance WMC and EFs in the early years of life (4). Furthermore, in the majority of cases, studies on WMC and EFs interventions involving pre-schoolers with autism propose activities that do not entail use of digital technologies [e.g., (18, 19)]. Cai et al. (18) propose a 12-week mini-basketball training program for 18 pre-schoolers with autism; following the intervention, the subjects exhibited significantly better performance in working memory and regulation as compared to a control group. A recent study conducted by Zhang et al. (19) sought to investigate the impact of a three-game intervention on EFs involving different groups of pre-schoolers with autism. Twenty-four pre-schoolers with autism were selected and divided into three groups; these groups took part in an eight-week programme of, respectively, sports games, pretend play, and comprehensive games. The authors found that the children involved in the sports games group and in the pretend play group significantly improved working memory and cognitive flexibility and comprehensive game group improved the working memory and cognitive flexibility, and the improvement of inhibitory control has reached a marginal significant level; furthermore, the intervention effect of comprehensive games was better than that of single sports games or pretends play.

To the best of our knowledge, only one study has been conducted that proposes an intervention featuring use of digital materials (20). This involved 24 children of preschool age. Specifically, the authors proposed an intervention in which a computer-based puzzle game named “Tatka” was employed; this was accompanied by a set of home-based tasks designed to enhance set-shifting ability. When comparing outcomes from pre- and post-test phases and from a repetitive behavior task, the authors found a significant change in both cognitive and behavioral flexibility. Furthermore, the result was to some extent sustained for about a month after the treatment.

So there is a clear paucity of available research on promotion of WMC and EFs in pre-school children with ASD using a variety of means, including digital applications. To fill this gap, the authors of this paper have conducted a study in which a playful digital-analogical rehabilitative intervention was conducted with a pre-school child with ASD. The study in question was carried out as part of the ShareFUN project.^1^ This follows the authors’ previous research (21), which has demonstrated that the integration of digital and analogical materials in interventions for preschool children could yield added value; this is because the two forms offer different affordances in clinical intervention contexts (22). For the digital activities, we opted for educational apps because they are low cost, familiar, and intuitive for pre-schoolers to use (23). What’s more, they have been proven to be fun to use, and motivating (24).

## Case description

For the intervention, one male child with ASD was selected from a group of ASD children attending a rehabilitation centre. This child met all the predefined inclusion criteria: (a) diagnosis of ASD; (b) age range 3–5 years old; (c) mild or moderate intellectual disability^2^; (d) deficit in WMC and EFs^3^; (e) the family’s willingness to participate in this study. To protect the participant’s identity, an assumed name (Francesco) was assigned, and all identifying information was removed from the study material. Francesco was 42 months old when his involvement in the study commenced.

### Family background and history

Francesco’s mother is a 29 year-old freelancer with a degree in communication sciences, while his father is a 28 year-old bar worker with a high school diploma. Francesco lives with his mother; his father had left the family and was not a party to Francesco’s clinical evaluation because he did not agree with the need for undertaking a diagnostic process (he did however provide the proxy to be able to carry out the diagnosis). Francesco’s maternal grandparents support the mother in carrying for him. The paternal family has a history of epilepsy and dyslexia, while the maternal family had a history of thyroid pathologies.

Francesco was born at the due date (spontaneous delivery) following a normal pregnancy: birth weight 3.34 kg, height 41 cm, good adaptation to extra-uterine life (Apgar not available). His mother opted for formula feeding and he had no difficulty in weaning.

Currently no sphincter control; regular sleep–wake rhythm.

Motor development: sitting independently at 7–8 months, walking autonomous at 15 months.

Language development: babbling at 9 months, first words at 28 months (during the diagnostic assessment period Francesco produced about 60 words, mainly in echolalia, and did not make standard hand gestures, e.g., when greeting.

At 16 months he started nursery school. At that time he also started psychomotor and speech therapy treatment.

### Diagnostic assessment

At 31 months, Francesco received a diagnosis of neurodevelopmental disorders, specifically ASD.

Adaptation to the evaluation context took place without particular difficulties. Spontaneous relational initiative was mostly directed toward the mother, whom the child sought above all for requesting purposes. Eye contact was not always appropriate. Francesco also had behavioral rigidity and performed ritual behaviors. Assessment with the Griffith-III developmental scale [Association for Research in Infant and Child Development (26)] revealed an immature level of development (limit area). Adaptive behavior assessment performed via VABS-II interview with the mother (27) showed a globally adaptive level in line with age group (Adaptive Behavior Composite Score = 97). The profile was homogeneous but at the same time some difficulties emerged: “Communication” = 80 and “Motor Skills” = 81 levels are considered below the norm and “Daily Living Skills” = 87 and “Socialization” = 86 levels are in the lower limits of the norm. Assessment with the ADOS-2 (28) returned a score above the cut-off for ASD. To investigate language competence, the MacArthur questionnaire (29) was administered. This revealed the following results: (i) “producing gestures” and “receptive syntax” as for a 17 month-old child; (ii) receptive vocabulary as for a 20 month-old child; (iii) expressive vocabulary as for a 23 month-old child. The Child Behavior Checklist [CBCL; (30)] filled in by the mother to detect any behavioral problems revealed a borderline level for deficit in the “Attention” area. The RBS-R questionnaire (31) to investigate repetitive behaviors did not reveal significant results but at the time of diagnostic evaluation, the mother reported the presence of some repetitive behaviors (opening and closing doors, turning switches on and off) and rituals (closing the school gate). The questionnaire Toddler Sensory Profile 2 (32) showed atypical levels in the “Seeking,” “Visual processing,” and “Movement” scales. The EDQ questionnaire (33) for detecting precocious development did not reveal developmental regression.

In summary, Francesco received a diagnosis of neurodevelopmental disorders, specifically ASD. His clinical profile revealed global immaturity in development, difficulty in the socio-communicative domain, atypia in the “interest” area, language disorder in both expressive and receptive domains, and motor hyperactivity.

## Materials and methods

### Procedure

Initially, two CNR-ITD researchers working jointly with a staff clinician from the rehabilitation center analyzed the inclusion criteria and selected Francesco as a suitable child with ASD diagnosis to involve in this study. As Francesco was a pre-school child, we chose a play-based rehabilitative intervention. Furthermore, considering the child’s specific deficit, all the activities we proposed were designed to promote WMC and EFs; these involved interaction with both analogue and digital materials, a feature intended to maintain Francesco’s level of engagement. Furthermore, considering Francesco’s nature in the socio-relational domain, we opted for a rehabilitative intervention that featured interaction between child and clinician.

Before commencing the treatment, one of the researchers trained the clinician in how to administer the designated cognitive tasks and conduct the rehabilitative intervention.

**Figure 1:**
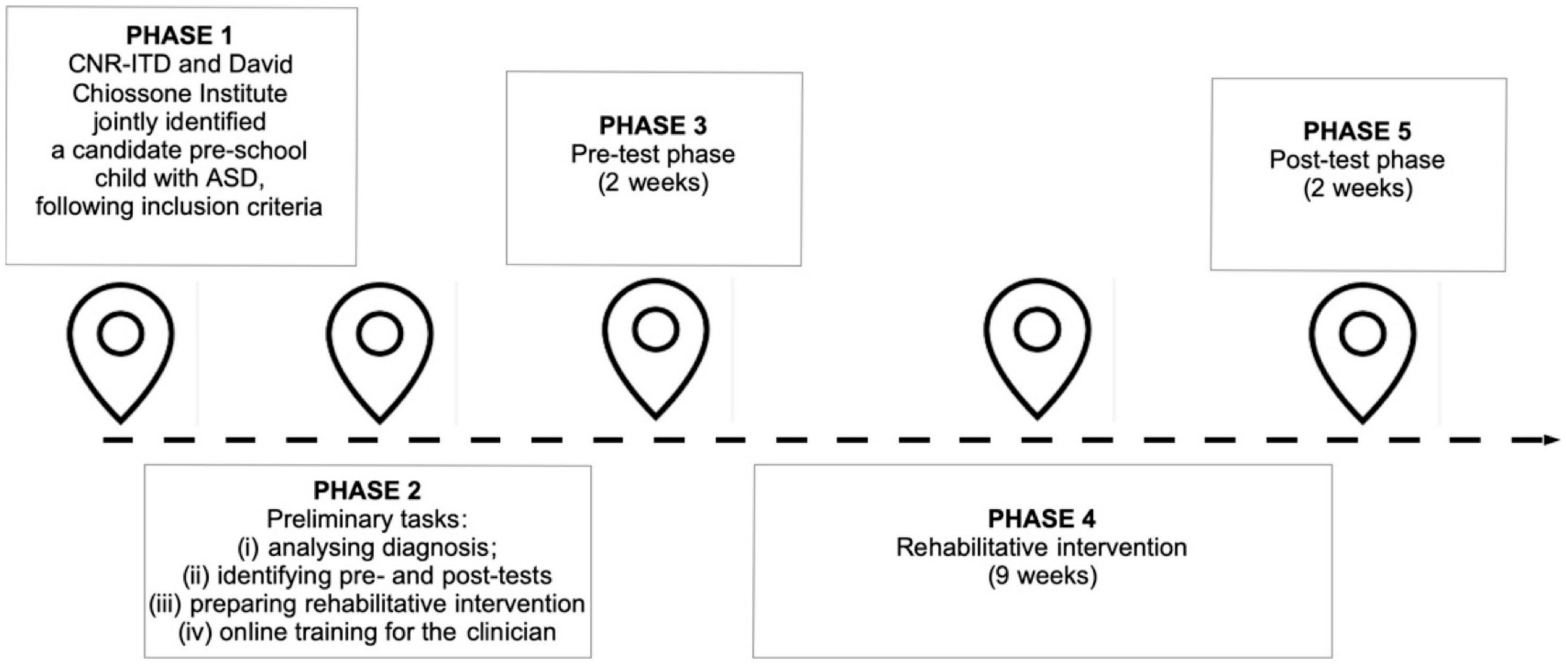
Timeline.

In the baseline phase, the clinician administered a series of cognitive tasks (see “Measures”). Subsequently, Francesco underwent 17 rehabilitative sessions, held twice a week for 9 weeks. Over this period, the clinician recorded the rehabilitation activity scores and noted qualitative observations. In the post-test phase, the clinician re-administered the cognitive tasks (Figure 1).

The case notes, observations, activity scores, and pre- and post-test results were all analyzed to assess the impact of the rehabilitative intervention and possible variables contributing to change.

### Rehabilitative intervention

As mentioned, the proposed intervention adopted a play-based approach in order to engage Francesco and maintain his motivation to collaborate.

The main aim of the opening session was to present the intervention to the child. The clinician proposed a doggerel to introduce the intervention program, using a sheep puppet and a visual agenda to explain the activities. During this session, the clinician established a pact with Francesco and designated a set of behavioral rules.

Francesco subsequently underwent rehabilitative sessions of three different kinds (sessions A-B-C); these were repeated five times each for a total of 15 sessions lasting 45 min each. To track Francesco’s progress in automation and/or improvement in the targeted cognitive skills, the clinician annotated activity performance scores on a registration sheet.

Each session followed the same core phase structure: (1) familiarization; (2) cognitive training; and (3) metacognition (for details, see Supplementary material).

In the familiarization phase, the clinician introduced Francesco to the daily activities using the Sheep Puppet Companion, proposing the doggerel and showing the main activities of the day using the visual agenda.

The cognitive training phase comprised four mini-games, two of which were analogue and two digital. Specifically, this phase was split in two sections. The first involved two playful memory games (a specially-created analogical game and an educational digital app game) to enhance short-term memory and WMC. These were followed by playful EF games, once again involving a specially-created analogical game and an educational app game. The predetermined game order (first analogical then digital) was adopted as Francesco considered playing with the app^4^ to be a reward. The decision to adopt the four short (3–5 min) mini-games, involving both analogue and digital materials, was driven by the intention to maintain interest, motivation and attention.

In the last phase, the clinician engaged Francesco in a metacognitive activity in which they reflected jointly about the strategies used in the games. In this phase, a set of visual strategy cards was employed.

In the last session, Francesco reflected with the clinician about all the activities undertaken. This included reflection about the strategies Francesco had used (metacognition). He then received a diploma of merit (prize) and a party was held involving the clinician, Francesco and his mother.

### Measures

To investigate the effects of the proposed playful analogical-digital rehabilitative intervention, various cognitive measures were adopted during the pre- and post-phases.

*Mr Cucumber* (34) – task for assessing WMC in the visuo-spatial domain. The silhouette of an extra-terrestrial figure was displayed for 5 s with one or more colored stickers attached. Following each display (game item), the child had to show the position/s of the sticker/s on a separate bare silhouette. The game had eight levels (from 1 to 8 stickers appearing in each) and each level comprised three silhouette-display items. One point was given for each level at which the child got at least two of the three items correct, and an extra third of a point was assigned when response to all three items was correct (range: 0–8).

*Backward Word Span* [BWS; (35)] – task for assessing WMC in the verbal domain. The child was required to repeat lists of words (ranging from 2 to 7 words) in reverse order. Three list-items were presented at each level. One point was given for each level at which the subject got at least two of the three items correct, and an extra third of a point was assigned when response to all three items was correct (range: 0–7).

*Direction Following Task* [DFT; (36)] – task for assessing WMC in the receptive language domain. This task required the subject to follow oral directions of increasing complexity. There were three levels, with five items presented at each level. The score was calculated following Morra et al. (37) and the adapted version for pre-schoolers [(17); range: 0–3].

*Day/Night Stroop* (38) – task for assessing the ability to inhibit an inappropriately verbal response and to activate an alternative. In the first phase, the subject was required to say “day” when shown a white card with a yellow sun, and “night” for a black card with a moon and stars. In the second phase, the subject was required to invert the card/word association. There were 16 items for each phase (range: 0–32).

*Simon Says* (39) – task for assessing motor inhibition. In the first game, the child is instructed to perform an action only when the verbal cue “Simon says” is pronounced immediately before the corresponding command is given (activation trial), and to refrain from carrying out that action if the cue is *not* pronounced first (inhibition trial). In the second phase of the game, an additional difficulty factor is added, namely the examiner performs each action regardless of whether the “Simon says” cue is pronounced or not. There were 10 items per phase. For each item, two points were given for the correct response, one point was given when the child self-corrected and inhibited his behavior, zero point was given when the child is wrong (range: 0–40).

*Circle drawing* (40) – task for assessing the ability to control ongoing motor response. It involves using a cardboard square with an 8.5 cm circle drawn on and a small arrow indicating the starting point. In baseline condition, the child moved a doll around the circle, and in a second condition had to repeat this with a toy snail, moving it as slowly as possible. The score was calculated as the proportion of the slowdown to the total time in both conditions using the following formula (T2 − T1)/(T1 + T2).

*Dimensional Change Card Sort* [DCCS; (41)] – task for assessing complex shifting. The child was shown a deck of cards with two variants – shape (rabbit, boat, etc.) and color. During the pre-switch phase, the child sorted the cards according to shape (6 items), and in the post-switch phase according to color (6 items). In the third phase, the experimenter explained that if a card had a black border, then the child had to sort according to shape, and if not, according to color (12 items). The pre-switch and post-switch phases were scored one point if at least five responses out of six were correct, and the border phase was scored one point if at least nine out of 12 were correct (range: 0–3).

*Magic House* (42) – task for assessing the constant monitoring and addition or deletion of working memory contents (updating). For each item, the child was shown three, four or five toy animals, which were placed sequentially in a cardboard house. He was then required to recall the last two animals placed in the house. There were nine items, each scored from 0 to 2 points (range: 0–18).

*Peabody Picture Vocabulary Test, third version* [PPVT-III; Italian version: (43)] – task for assessing receptive vocabulary. The experimenter read a word, and the child was asked to select the corresponding picture from a set of four. If the child gave eight correct responses before the first error, a ‘basal’ was established. The task then continued until the child reached an error rate of six out of the last eight items (ceiling). The score was the sum of correct responses; the items before the basal were considered correct (range: 0–175).

## Results

The child was able to perform all the proposed digital and analogical activities, indicating the intervention’s feasibility. He participated in both types of activity with interest and enthusiasm, demonstrating a preference for the apps.

**Figure 2:**
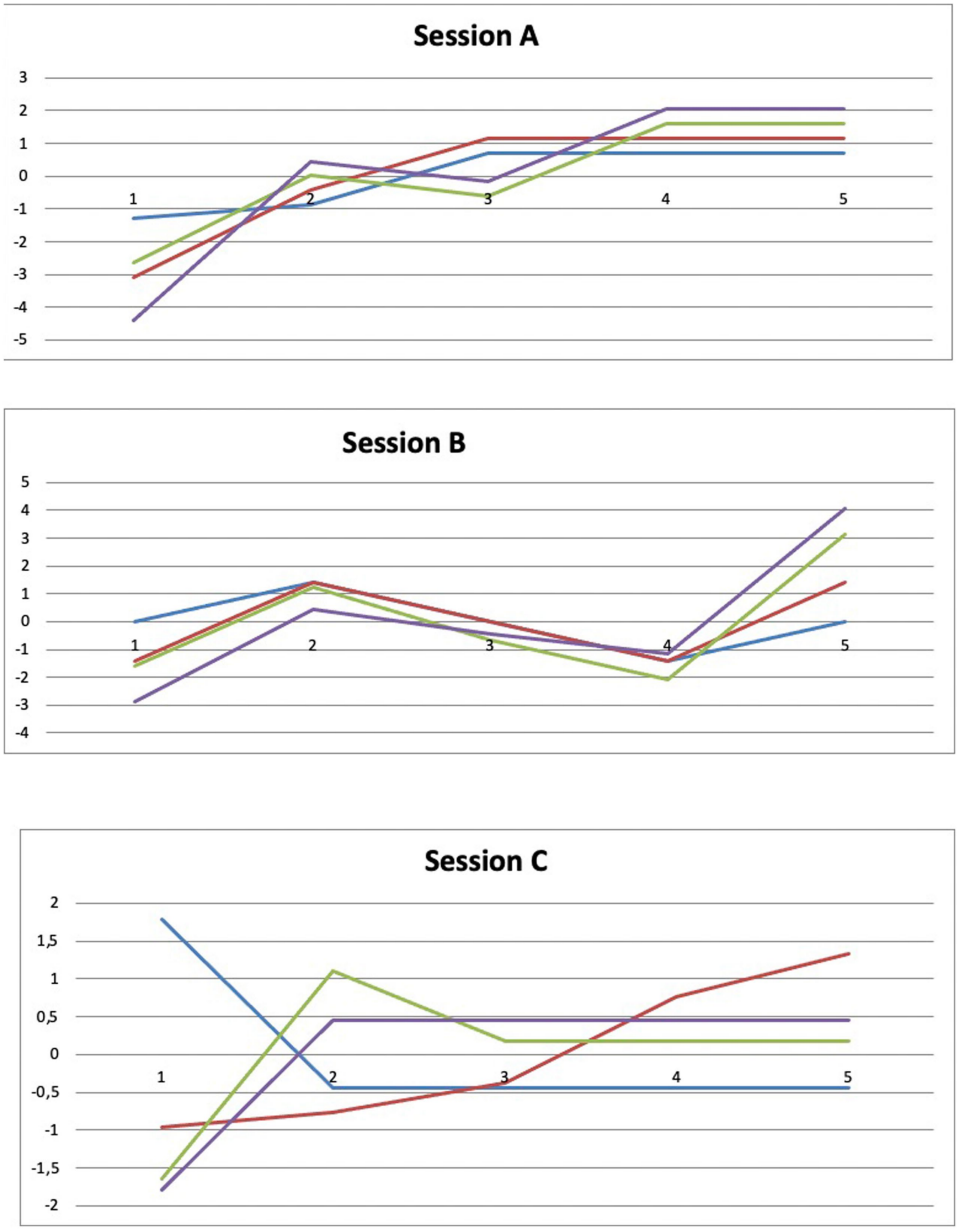
Changes in activity performance through the rehabilitative intervention (z-points). Blu lines: performance in analogue memory games; Red lines: performance in digital memory games; Green lines: performance in analogue EFs games; Violet lines: performance in digital EFs games.

We transformed the raw scores from each activity into z-points. Figure 2 presents a visual representation of Francesco’s performance changes during the intervention. This shows qualitative improvements in almost all activities, especially those with apps. His progress in all sessions represents positive consolidation of abilities and achievement of the intervention goals.

In addition, comparison of pre- and post-assessments revealed some qualitative improvements. Concerning WMC, a small improvement in the DFT was found (from one to two correct responses at Level 1).

As to inhibition, the Circle Drawing task revealed an important finding: if we analyze only time, it seems that Francesco’s performance declined, passing from performance between the twenty-fifth and fiftieth percentile to performance under the fifth percentile (44). However, when considering accuracy, we observe that in the post-test phase he did not make any errors. So, Francesco’s performance declined in terms of time but improved in accuracy. A small inhibition improvement was also detected in the Simon Says task (from 16 to 20). Another small improvement was also detected in the updating task, Magic House (from 8 to 9): in the pre-test phase, Francesco obtained a score between the tenth and twenty-fifth percentile, while in the post-test phase between the twenty-fifth and fiftieth percentile (42). Finally, a qualitative improvement was also detected in the receptive language task, PPVT-III (from 7 to 17). In line with Stella et al. (43), in the pre-test phase Francesco had a weighted score of 67 and in the post-test phase a weighted score of 73.

In those tasks in which the child achieved better scores in the post-test phase, we also utilized the Jacobson and Truax (45) approach to reliable clinical change including calculation of the Reliable Change Index (RCI >1.96) using previous data sets to obtain the standard deviation and α coefficient. The RCI is a statistic that determines the magnitude-of-change score necessary for a given measure to be considered statistically reliable.

For the Simon Says activity, the RCI was calculated using the SD (7.39) and α coefficients (0.82) from a large sample of pre-schoolers (46).

The RCI for the DFT score was computed using the SD (1.08) and α coefficients (0.84) for the total score from a large sample of pre-schoolers (17).

The RCI for the total score of the Magic House activity was computed using the SD (2.12) and α coefficients (0.72) for the total score from a large sample of pre-schoolers (42). Specifically, SD regarded a subsample of children from 36 to 48 months old.

For the PPVT-III, the RCI was calculated using the SD (21.20) and α coefficients (0.96) from a large sample of pre-schoolers (47).

Although qualitative changes emerged from pre- and post-test assessment, significantly reliable improvements were not noted in Simon Says (RCI = 0.90), Magic House (RCI = 0.63), DFT (RCI = −0.54) or PPVT-III (RCI = 1.67), even if the last one approached statistical significance (Table 1).

**Table 1:** Pre- and post-assessment scores and RCI index.

Measure	Pre	Post	+ improved; = no change; − worse	RCI index
Mr Cucumber	1	1	=	
BWS	1	1	=	
DFT	0.33	0.66	+	−0.54
Day/Night Stroop	31	27	−	
Simon Says	16	20	+	0.90
Circle Drawing Time	0.12	−0.31	−	
Circle Drawing Accuracy*	It goes around randomly leaving and reentering the circle	No error	+	
DCCS	2	2	=	
Magic House	8	9	+	0.63
PPVT-III	7	17	+	1.67

*For the circle drawing time data are available to compare findings with the normative sample [see (44)] but not for accuracy. We reported here data about accuracy as qualitative information.

## Discussion

This study provides preliminary support for the feasibility of a new playful digital-analogical rehabilitative intervention for children with ASD, starting from pre-school age. The experience gained within this study gave us direct and concrete understanding of some key positive and critical aspects.

### Key positive aspects of the intervention

On the positive side, the intervention set-up was well suited to the study’s objectives; the tablet proved to be a very attractive device for promoting interest, motivation and attention. Indeed, while Francesco was very happy to carry out both the digital and analogical activities, he preferred those with the educational apps. During these activities, he showed greater motivation and stronger improvement across the various sessions than in the analogue activities. This is consistent with findings reported in the literature, which suggest that pre-school digital-native children are very attracted and motivated by apps, a means they find very familiar and intuitive (24). Furthermore, the study findings also confirmed the capacity of educational apps to enhance WMC and EFs in an intervention program (21, 48). This is also in line with studies involving children with ASD, which highlight the added value of using digital technologies during the intervention (14), in particular when seeking to enhance cognitive abilities such as WMC and EFs (4). Overall, Francesco was able to tackle all the activities, both analogue and digital, and improved in performance. These findings underline the feasibility of the playful digital-analogical rehabilitative intervention proposed.

Analysis of pre- and post-assessments reveals qualitative enhancements in some cognitive domains. Specifically, a small improvement emerged in WMC. Concerning inhibition, we note enhanced accuracy in two tasks requiring inhibition in the motor domain; however, improved accuracy in the Circle Tracing task was offset by slower times. One very important finding regards improvement in Francesco’s updating ability, an EF construct that remains both under-investigated and controversial where pre-schoolers are concerned (12), especially with ASD (9). These qualitative changes were not confirmed by the statistical analysis. This result could indicate that the intervention does lead to improvement, but to achieve significant improvement the intervention would need to continue over a longer time span.

The intervention not only seems to yield small qualitative improvements in WMC and EFs (the cognitive abilities directly enhanced), but also in language in the receptive domain, where it approaches statistical significance. This finding demonstrates that, when pursuing enhancement of WMC and EFs, interventions can also boost language capabilities, which, in the pre-school period, are related to WMC and EFs (49).

Furthermore, it is worth noting that the intervention featured opportunities for metacognitive reflection. This gave Francesco the chance to reflect jointly with the clinician about the strategies he adopted to undertake the various activities. This reflection was conducted using visual strategy cards, and this could have heightened awareness of his specific cognitive style and how it impacts on his daily functioning.

### Limitations and further research

The findings reported in this study need to be considered in the light of the inherent limitations of a case study. Further studies on larger samples are needed to gain more comprehensive evaluation of the feasibility and generalizability of this intervention with children with ASD. Future studies with ASD children should seek to include both an experimental group that undergoes the intervention and a control group that does not. This is ambitious due to the heterogeneity of children with ASD. However, given that the playful digital-analogical rehabilitative intervention is customizable, it would be possible to investigate whether it could also be proposed to children along the spectrum of the autism. Another important aspect to be considered is that the pre-school child in the study only managed to pass the first levels of the proposed activities. This could mean that these activities are overly challenging for pre-school children. Therefore, we believe that it would be more appropriate to engage school-age children with ASD who have a medium or high cognitive level. Consequently, further research is needed to explore the actual feasibility and effectiveness of the intervention with school-age children with ASD as well.

### Suggestions for clinical practice

The present case study provides some suggestions for clinical practice. First, in order to improve WMC and EFs in children with ASD, it’s important to propose activities with a number of key characteristics so as to engage the subject and maintain motivation. Specifically, they need to be playful in nature, brief in duration, feature levels of progressively increasing difficulty, and involve interaction with a variety of materials, both analogue and digital. In this last case, it’s preferable to propose analogue activities first and then move on to digital activities, which may be perceived by the child as a kind of reward. Furthermore, it’s important to use mainly visual materials, since this is the preferential channel for most children with ASD, many of whom speak little or even not at all.

Furthermore, it’s worth noting that in this case study the subject did obtain qualitative improvements – as revealed by results from the pre- and post-test phases - but only in some cases did those improvements approach statistical significance. With this in mind, it is advisable that future research should propose longer interventions as children probably need more experience with a greater number of activities in order to enhance WMC and EFs. Finally, where possible, parental involvement is important as children’s motivation is boosted when they know they can show their progress to their parents at the end of the intervention.

## Data availability statement

The raw data supporting the conclusions of this article will be made available by the authors, without undue reservation.

## Ethics statement

Ethical approval was not required for the study involving human samples in accordance with the local legislation and institutional requirements. Written informed consent for participation in this study was provided by the participants’ legal guardians/next of kin. Written informed consent was obtained from the individual(s), and minor(s)’ legal guardian/next of kin, for the publication of any potentially identifiable images or data included in this article. Written informed consent was obtained from the participant/patient(s) for the publication of this case report.

## Author contributions

SP: conceptualization, methodology, investigation, data curation, formal analysis, and writing—original draft. MD: collecting information about the patient’s condition and data curation. LF: project administration, Funding acquisition, supervision, and writing—review. All authors contributed to the article and approved the submitted version.

## Funding

This study was conducted as part of the ShareFUN project, a co-funded project supported by the Operative Program Por FSE and Liguria Region 2014–2020, code: RLOF18ASSRIC/77/1.

## Conflict of interest

The authors declare that the research was conducted in the absence of any commercial or financial relationships that could be construed as a potential conflict of interest.

## Publisher’s note

All claims expressed in this article are solely those of the authors and do not necessarily represent those of their affiliated organizations, or those of the publisher, the editors and the reviewers. Any product that may be evaluated in this article, or claim that may be made by its manufacturer, is not guaranteed or endorsed by the publisher.

## Footnotes

^1^The ShareFUN project (www.sharefun.it) entailed various interventions designed to enhance WMC and EFs in children with neurodevelopmental disorders and/or from low Social Economic Status (SES) backgrounds.

^2^The intellectual disability level was set at mild or moderate because this type of intervention is too difficult for pre-schoolers with a high level of intellectual disability to understand or follow.

^3^In the selection phase, we consulted clinicians who had carried out various activities with the chosen subject. They reported that he had difficulty performing various activities that entail memorizing information, paying attention to tasks, and controlling behaviour. We also consulted the mother, who reported the same information as the clinicians. We invited the mother to complete an observation survey, BRIEF-P (25), for screening purposes. The mother reported that her son found it particularly difficult to perform activities involving WMC and inhibition.

^4^For details about the educational apps used in the rehabilitative intervention, see Supplementary material.
