# Supplementary material for: Case Report: A playful digital-analogical rehabilitative intervention to enhance working memory capacity and executive functions in a pre-school child with autism
Source: Front Psychiatry. 2023 Sep 29;14:1205340. doi: 10.3389/fpsyt.2023.1205340 (PMC10570721; doi:10.3389/fpsyt.2023.1205340)
Supplement: Supplementary file 1 [file Data_Sheet_1.pdf]

Supplementary Material

Case Report: a playful digital-analogical rehabilitative intervention to enhance Working Memory Capacity and Executive Functions in a pre-school child with autism

Sabrina Panesi*, Marina Dotti, Lucia Ferlino

***Correspondence: Sabrina Panesi:** panesi.sabrina@gmail.com

Supplementary Figure 1. Structure of the A-B-C sessions

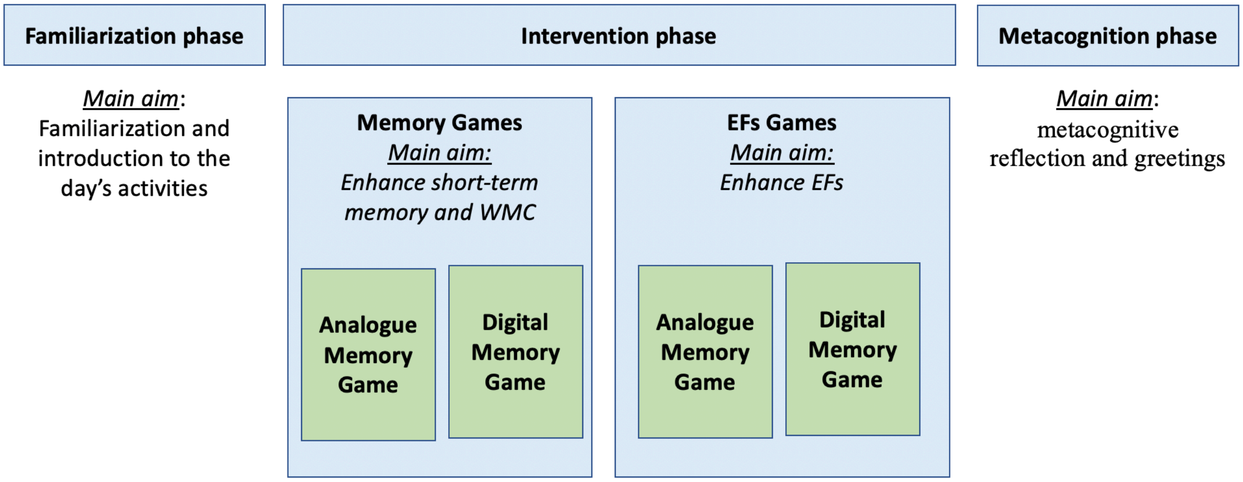

Supplementary Table 1. Intervention with analogue games

Intervention Phase	Session	Activity description	Example
**Memory game**	A	In this game, the child has to memorize a sequence of animal cards. To start off, the clinician presents two animal cards. The child has to memorize the sequence of animals and then place the cards in the correct order in some circles. The clinician shows three card sequences.<br>If the child answers correctly to a two-animal card sequence, the number of animal cards presented is increased; conversely, the game is terminated.	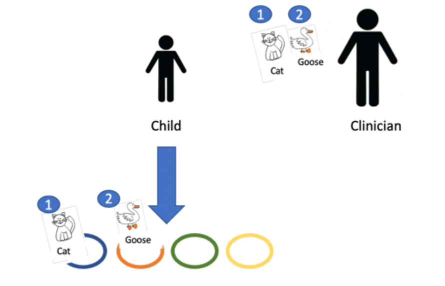
	B	In this game, the child has to memorize the sequence of animal cards in reverse order. When the clinician presents the first two animal cards, the child has to memorize the animal sequence and then place them in some circles but in reverse order. The clinician shows three card sequences.<br>If the child answers correctly to a two-animal card sequence, the number of animal cards presented is increased; conversely, the game is terminated.	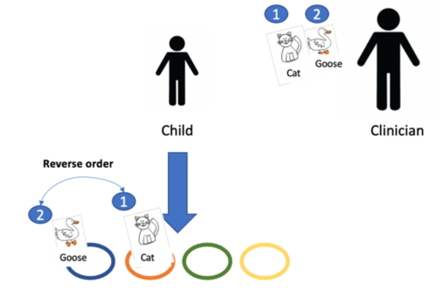

<table>
<tr><td></td><td>C</td><td>In this game, the child sees a series of cards depicting a sheep with one or more superimposed marks. The child has to memorize these and subsequently indicate the same marks on a blank sheep. At the first level, a sheep with one mark is shown, increasing to three at level 3. If the child answers a level correctly, they progress to the next level; conversely, the game is terminated.</td><td>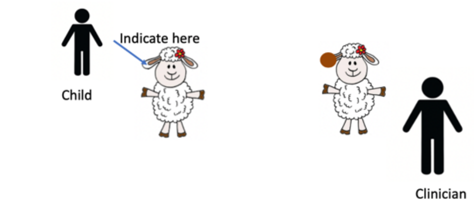
</td></tr>
<tr><td rowspan="2">EF game</td><td>A</td><td>This game has three levels. At level 1, the child sees a series of cards depicting different green animals. When a card is shown, the child first has to say the colour “green” and then the name of the animal (e.g., cat, rabbit). At level 2, green or red animals are presented. Only when a green animal appears should the child nominate the colour and the animal. At level 3, the child sees a series of cards depicting animals (red or green) or vegetables (red, green or orange). The child has to nominate the colour (red, green or orange) of a vegetable card, and the name of the animal (e.g., cat, rabbit) for an animal card.</td><td>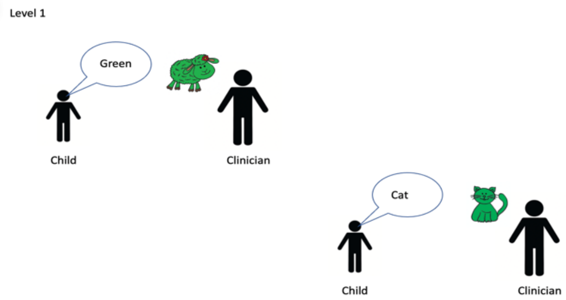
</td></tr>
<tr><td>B</td><td>In this three-level game, the child has a series of five circles in front of them on the floor, and the clinician has a red/green ‘stop-go’ light. At level 1, when the green light comes on the child has to jump into the circle immediately in front of them but must stop when the red light comes on.
At level 2, the child sees a series of red or green animals. The child has to jump into the circle in front of them only when they see the green sheep. At level 3, the child sees a red or green light and subsequently an red or green animal card. The child has to jump into the circle in front of them only when the green light appears and a card depicting the green sheep is shown; in all other cases, the child has to stop.</td><td>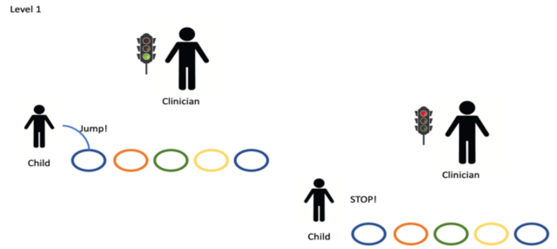
</td></tr>
</table>

	C	In this game, there are three levels. At level 1, the child has to put cards depicting small animals in a small box and cards depicting large animals in a large box. At level 2, the task is inverted: large animal cards go in the small box and small animal cards in the large box. Level 3 reverts to the level 1 task except that there is a pink ball on some of the cards. When a pink-ball card is shown, the child has to perform the inverted level 2 task. i.e. put large animal cards in the small box and small animal cards in the large box.	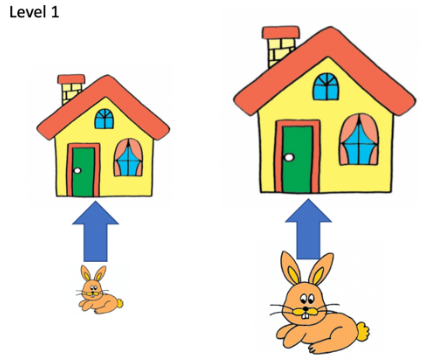

Supplementary Table 2. Intervention apps

Intervention Phase	Session	App used	Link	Activity description	Screenshots
**Memory game**	A	Kids Educational Games: Preschool and Kindergarten – Auditory Memory	Android: https://play.google.com/store/apps/details?id=air.com.shubi.LearnCNLW.english&hl=it	In this game, the child hears a series of sounds coming from some monsters and then an on-screen loudspeaker reproduces one of the sounds the monsters made. The child has to identify the monster that made that sound by touching it. If the child answers correctly, the number of sounds the monsters make increases; conversely, if the child makes a mistake, the number of sounds decreases.	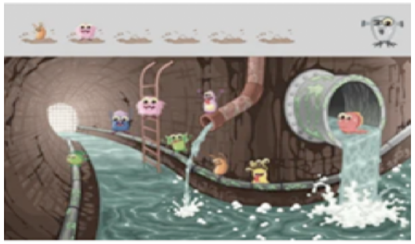

	B	Masha and Orso – educational games	Android: https://play.google.com/store/apps/details?id=com.edujoy.masha.games&hl=it&gl=US	In this game, the child has to memorise a sequence of colours and then reproduce it. Initially, three colours are presented and one lights up; the child has to memorize this and then click on it. If the answer is correct, the set of colours presented increases, as does the number to memorise (2 then 3). If the child continues to answer correctly, the game automatically passes on to the next level, (four colours presented), and so on.	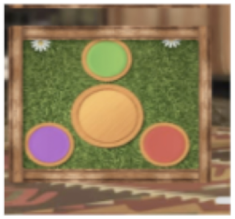
	C	Masha and Orso – educational games	Android: https://play.google.com/store/apps/details?id=com.edujoy.masha.games&hl=it&gl=US	In this game, a given set of images is presented for the child to memorize. Then the set is presented again but this time with a new image included. The child has to recognize and tap [or click – see above] this .At the beginning, the set comprises two images; if the child identifies the new addition correctly, the number of pictures to memorize increases.	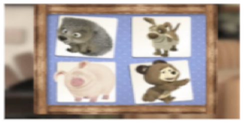
**EF game**	A	Bimi Boo educational apps	Android: https://play.google.com/store/apps/details?id=com.bimiboo.playandlearn&hl=it	In this game, the child is shown animals and objects of different colours. First these are to be placed in train carriages of the corresponding colour. Subsequently, the train arrives in front of different houses in those same colours, and the child has to correctly position the animals and objects, classifying them by colour.	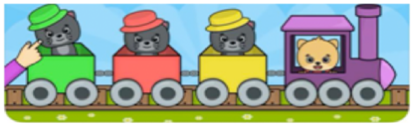

	B	Masha and Orso – educational games	Android: https://play.google.com/store/apps/details?id=com.edujoy.masha.games&hl=it&gl=US;	In this game, a set of rabbit holes is shown, from which rabbits of different colours emerge in sequence. The child is only supposed to tap a grey rabbit when it appears.	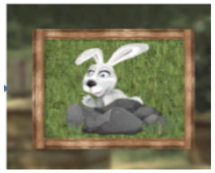
	C	Bimi Boo educational apps	Android an iOS: https://play.google.com/store/apps/details?id=com.bimiboo.playandlearn&hl=it	In this game, the child has to classify a set of objects according to their size.	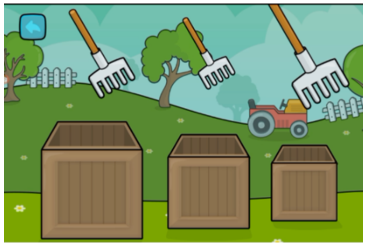
